# Lord Crewe on National Insurance and the Voluntary Hospitals

**Published:** 1913-01-18

**Authors:** 


### LORD CREWE ON NATIONAL INSURANCE AND THE VOLUNTARY HOSPITALS.

### Opening of the New Wing at Beckett Hospital, Barnsley.

On Thursday last week the Marquis of Crewe formally opened a new wing at the above hospital. In consequence of the latest additions to this institution, which was founded in 1864, and has from time to time been improved, by the Marshall Wing, for which the late Mr. J. L. Marshall, of Monk Bretton, bequeathed £5,000, and by a new operating theatre presented by the Barnsley Co-operative Society at a cost of nearly £2,000, it is claimed that the institution is now one of the best equipped of its kind in South Yorkshire. We are indebted to the Editor and staff of the *Sheffield Daily Telegraph* for the report which follows.

The new portion comprises accommodation for thirty-six additional beds, making a total of 108 beds, a rearranged out-patients' department, electric lift, eye department with x-ray room, kitchens, electric laundry, board room, together with complete post-mortem room and mortuary. The entire cost of this means an expenditure of nearly £18,000, for which the Building Fund shows a deficit of nearly £4,000. The Marquis of Crewe was the guest of the Hospital Committee at a luncheon prior to the inspection of the institution and the opening of the new wing, which is already occupied. A public reception followed in the Public Hall, where a large gathering was presided over by Mr. C. J. Tyas, M.A., who was supported by the Mayor of Barnsley, Councillor J. H. Cotterill; the Bishop of Wakefield; Sir Henry Burdett; Mr. Alfred Whitham, vice-chairman of the House Committee; Mr. Hy. Feasby, finance chairman, Saturday and Sunday Fund; Major Thelwall Maurice, R.A.M.C.; the medical staff of the hospital; and Mr. R. F. Pawsey, honorary secretary of the hospital, with other gentlemen.

### The Marquis of Crewe's Speech.

Lord Crewe said : I cannot pretend to be altogether a stranger in Barnsley, but that does not prevent me from thanking you—indeed, it further induces me to thank you—for having done me the honour of asking me here to-day to celebrate the opening of this important addition to the Beckett Hospital. When I first knew Barnsley—now nearly thirty years ago—I used to come here on a somewhat different errand, the errand of a political candidate, which appeals always to some parts of an audience such as this, but by no means appeals to the others. On this occasion, however—and this is what makes the day and moment so pleasant to me—I am here on behalf of a cause which appeals to every man and woman in the district. The well-being of this hospital is, as I well remember it was in the old days, most dear to the whole population of Barnsley and neighbourhood. It is the charity of all others which I think is nearest to the hearts of the people of Barnsley and district. That I say without any fear of contradiction. The Chairman has told us how it arose from humble beginnings, from the useful but unambitious functions of a dispensary. It gradually grew, and in 1884 one of the principal wings was added. Since then it has gone on from strength to strength until we see the fine, commodious, and complete building which we have just visited this afternoon, replete with all the modern inventions of science, and destined, as we hope, to alleviate pain and trouble, not merely for the people in the town of Barnsley, but for a considerable district around, in its present form for many years to come. The Chairman has told us how the Marshall Wing has been added largely through the munificence of the late Mr. Marshall. Then there is the important and most remarkably perfect addition of the operating theatre, with which I was greatly impressed.

The Spirit of the Neighbourhood.

That, I understand, is due to the wise forethought and liberality of the co-operators of the district. All these additions have involved an expenditure of something close on the very large sum of £18,000, and the net result, after all the benefactions that have been received towards it, leaves a debt of something approaching £4,000 upon the Building Fund. With that debt it is rightly desired to cope as soon as may be. I am told that there is a project for holding a great bazaar in the future, by which I should hope a large portion of the debt may be liquidated. Of one thing I am certain, that the interest in Barnsley and the neighbourhood in this institution is so great and so enthusiastic that men and women will work together to their very utmost for the purpose of wiping off the deficit and of insuring that the handsome subscriptions which are forthcoming year by year to the maintenance of the hospital can all go for the purpose of actual maintenance, and will not to any degree be taken up with the payment of interest on the existing debt. That, I am quite certain, will be the aim of everyone in the town, and I shall look forward to hearing before very long that the debt has been wiped away. Nobody, I think, who has looked at the recent reports of the hospital and its management can fail to be impressed by the steady growth, to an amount which, when one considers all the circumstances, is really amazing, of the Saturday Hospital Fund. It shows the predominant pride that is taken in the hospital that so large a proportion of its necessary expenditure is met by this vast aggregation of small contributions. These small contributions last year actually reached, as a result of one day's work, the sum of upwards of £1,000. That has the double value of releasing the large sum of money which is required for the maintenance of the hospital, and also of testifying to the human interest which each donor feels in the institution of which he is in a small way a supporter. That greatly adds to its value both as a remedial institution and as witnessing to the human feeling of the district.

### The Effect of Recent Legislation.

I am desirous of saying a word on one subject of current interest, because it is one which has greatly exercised the minds of many of those who are concerned with hospital work. That subject is this : What is the present effect and what is likely to be the future effect upon our great hospital system in Great Britain of recent legislation and in particular of the National Insurance Act? As I have every reason to know, the minds of many who are engaged in hospital work have been deeply exercised upon this subject. That has been shown both by frequent contributions to the daily press and also by important formal deputations which have visited members of the Government in relation to this particular branch of the effect of National Insurance. I need not reassure the company by stating that I am in no way going to discuss either the policy or the provisions of the Insurance Act. This is neither the time nor the place for such a disquisition. Still less am I going in the most distant manner to allude to certain differences of opinion, amounting in some cases to positive friction, which have grown out of various provisions of the Act. But I am desirous of saying one or two words which I hope may prove to be of a reassuring character about the Act in relation to the special subject of our voluntary hospital system. That system of voluntary hospitals is not only a great pride to us in Great Britain, but it is also, roughly speaking, peculiar to this country.

THE VALUE OF THE VOLUNTARY SYSTEM.

There is nothing on the continent of Europe in any way not merely precisely analogous to but even at all comparable with our great system of voluntary hospitals. That system, like the system of small contributions, is of double value. It not only provides valuable results in the healing of the sick, but an important result is to be seen in the great medical schools which cluster round the hospitals for the purpose of training medical students. Beyond those material gains we have the striking effect upon our national character and upon our social activities of the vast volume of voluntary work which is done by those who undertake the charge and management of hospitals all over the country. The value of that it is difficult to overrate, and when a deputation of Chairmen of London Hospitals came to see the Chancellor of the Exchequer in regard to some of the points on which I am going to speak, he most amply recognised the importance of maintaining the voluntary character of our great national hospitals.

It has, therefore, not merely been the desire of those who framed the Insurance Act, and the Government who were responsible for it, not to interfere with the prosperity of hospitals as we know them, but in framing the Act and passing it through Parliament it was their intention to take every care to avoid the inclusion of any provision which could possibly have the effect of injuring existing hospitals, either by diminution of subscriptions paid to them or in any other way. It cannot be contended that this is altogether an easy thing to achieve because we have to realise that as the ideas of mankind tend to an extension of the interference or the regulation of the State in matters affecting the well-being of the inhabitants of the country, as these ideas extend we find ourselves more and more confronted with the problem of how to combine that intrusion, if you like, of State management with the full and adequate maintenance of voluntary agencies. That problem has confronted us in a great many different forms, again, without distinction of political parties, on such diverse questions as the provision of education, the provision of small holdings in agricultural districts, and in the provision of old-age pensions. In all these subjects, none of which can be called party subjects, although party feelings have been allowed to grow warm upon all three of them—in all these subjects the problem has had to be faced of the proper limitation of State interference.

THE LIMITATIONS OF STATE CONTROL.

How far can such State interference be introduced without destroying the work satisfactorily carried on by voluntary agencies? The problem is how to maintain the interest and enthusiasm of those people who are prepared to give their time, their trouble, and sometimes their money for voluntary work, how to keep that side alive, while at the same time combining with it the greater perfection of organisation, that more complete covering of the ground, and that process of seeing that no corner is left undusted which can probably be carried out more perfectly by a State organisation than it can be by any organisation of a purely voluntary character. The problem, therefore, is one of great difficulty, and it is one which had to be faced in relation to this hospital question when the Government introduced the measure for enforcing a system of National Insurance. The way the question was tackled, or at any rate was intended to be tackled, was by keeping entirely separate and distinct the different forms and kinds of medical relief—that is to say, by devoting the system of National Insurance, while not entirely to the minor ills of life, at any rate to that class of medical relief which has hitherto been largely dealt with, and well dealt with, but not entirely dealt with, through the friendly societies and similar agencies and to leave alone, and to have no responsibility for, the whole system of hospital treatment, which it is desired to leave on its present voluntary basis.

**Hospitals Left Out of the Act.**

The moment the State takes part in the management of a hospital, or, still more, if it makes the taxpayers contribute to hospitals, it is bound to demand some measure of, at any rate, partial control. So far as I am aware, the one desire of the managers of our great hospitals is to be left altogether alone, and to have no touch or tinge of State control. And that being the object it was desired, through the provisions of the Act, to carry it into effect. Now, if we have succeeded, as I believe we have, in leaving hospitals altogether out of the Insurance Act, it surely follows that there is no reason whatever why the contributions of employers to hospitals should be diminished by the smallest fraction. Neither is there any reason why there should be a diminution of the small contributions paid by the workers, mainly insurable people. The latter are subscribers for certain benefits of a similar kind to those which have been received by them through benefit societies, through clubs, and trade societies, which subscriptions have no relation whatever to the possibility of a man having to go to a hospital.

Therefore, I confidently believe that it will be seen, and, indeed, that it is beginning to be seen, whether or not employers or employed consider the payment under the National Insurance Act a grievance, that it has absolutely no relation at all to the question of hospital subscriptions, and that the one ought not to be allowed to operate to the diminution of the other. What shows I am right in saying this is the fact that of the in-patients of the hospitals at this moment a very large proportion are insured persons. It is calculated, even in the great London hospitals, where the percentage must undoubtedly be smaller than it would be in a hospital like the one we have just visited, that some 40 per cent. of the in-patients are insured, or, at any rate, insurable persons. Clearly that shows what a large and continuing interest the insured persons and their employers have in the maintenance of hospitals, and it is on these figures possible to argue that it would be unreasonable for them to withdraw or even diminish their contributions. I have been told that two, at any rate, of the principal London hospitals have expressed their belief that so far as their subscriptions are concerned they have nothing to fear from the passing of the Insurance Act. I would very much like to hear what Sir Henry Burdett has to say on this subject, because it is a subject on which no one speaks with greater authority than he does.

The Hospital Point of View.

From the hospital point of view there are one or two ways in which hospitals as a whole ought to derive a certain degree of actual benefit from the passing of the Insurance Act. It is, of course, much if you are able to say they are not damaged by the Act, but I think it may be fairly argued that there are certain definite points in connection with this institution of National Insurance which are directly to the advantage of hospitals. In the first place, there ought to be in future, when a great medical system is fairly started under the auspices of the Act, a sensible relief in the number of people demanding admission as in-patients. Every doctor will tell you that among his hospital patients there is a considerable proportion who never, by the nature of their illness, ought to have had to go to a hospital at all, but through neglect their illness has developed in a manner which has changed it from a slight to a serious illness. If you can get a wider scope and a more scientific system of early treatment, as I hope will be the case when the Act comes into force fully, one of its effects should be a distinct diminution of the number to be admitted as in-patients.

Effect on Out-patients.

On the out-patients' side it, of course, cannot be denied that one effect of the establishment of a medical service contemplated by the Act will be the diminished number of hospital out-patients. How far that may be regretted by hospital managers I do not know, but it is clear that from a scientific point of view, regarding the hospital as part of a medical school, it is upon the in-patients that the function must necessarily rest of acting as educator of the students and the young doctors. I notice in the last report of this hospital the sum chargeable in respect of out-patients was put down at some £250 a year. I have seen it stated elsewhere that the cost is about 9½d. per head. Therefore, there would be a practical saving to the funds of a hospital in the diminution of out-patients. The most substantial change would be the saving of time to the medical staff.

### A Point of Importance.

Then there is one further point, and one of some importance, because it deals with a matter upon which the hospital authorities have been considerably exercised—viz., the arrangements which may conceivably be made, and in some cases are actually being made, between hospitals and approved societies for a kind of mutual accommodation. That is to say, that as the Act stands, if an insured person has no dependants and has to go to a hospital, the money payable to him is not required for the upkeep of the home, and therefore goes to the friendly societies. The hospital authorities have argued that in such cases it might very properly go not to the friendly society, but to the hospital that is looking after the man.

I think in logic, and even in sense, which is much better than logic, there is much to be said for the proposition. I am told there are cases, and I cannot help thinking there will be more cases, in which friendly societies, in consideration of accommodation being provided at need for their members who have sudden illness, or meet with an accident, and become candidates at short notice for admission to a hospital, that in consideration for such accommodation being always forthcoming the friendly societies would agree to subscribe either a universal sum or a sum per patient to the funds of the hospital. If so, some such arrangement would tend to console the hospital authorities for their position under the provisions of the Act and subdue any friction that might exist between them and the friendly societies.

Hopes for the Future.

I hope the general effect will be, and is beginning to be, that the disinclination to subscribe to hospitals and infirmaries, which for a short time was evident both among employers of labour and to some extent also among insured persons, is going to pass away. I do not think it is difficult to show that there is very little real basis or foundation for such an inclination. I cannot help thinking that when the first shock of a piece of legislation such as this has passed away—and new legislation almost always causes a shock, and the effect of such a shock is very often that people button up their pockets—when the effect has passed away, I for one am hopeful there will be no diminution in the amounts subscribed to hospitals and infirmaries, but, on the contrary, as the wealth of the country continues to increase, as it appears to be increasing at the moment by leaps and bounds, that these subscriptions will go on rising proportionately in the way that they have in the past. I thought it was due to myself as a member of the Government responsible for this Act of Parliament to endeavour to put before you some considerations which might make you believe that the Act, whatever its merits or demerits, at any rate was not merely framed with a desire to avoid hurting our great hospital system, but as framed and carried out it is actually succeeding in avoiding doing them any damage.

The Bishop of Wakefield, who, on behalf of the trustees, committee, and governors, moved a vote of thanks to the Marquis for his thoughtful and able speech upon an exceedingly difficult and intricate subject, said hospital work was one object upon which we could combine without distinction of age, class, or creed.

The Mayor seconded.

### A Scheme of Co-operation.

Sir Henry Burdett, who supported, did not altogether agree with the remarks of the Secretary for India regarding the effect of the Insurance Act on voluntary hospitals, and he outlined a scheme of co-operation which he commended to the consideration of the Government. He declared that if we were to lose the voluntary hospitals for the working-classes, who were so dependent upon them, the calamity would be so grievous that he did not know what would happen.

The Chancellor of the Exchequer had laid it down that medical benefit under the Insurance Act only meant minor treatment at home, and it was a most important point to bear in mind. He could not agree with the Marquis of Crewe's view that the Act would result in fewer patients applying for treatment at the hospitals.

More Patients Anticipated.

"I believe we shall have a very large increase in the number of persons who will apply for inpatient treatment, because you must remember the Insurance Act brings in fourteen million people who are to be attended for minor ills only by the doctors on the panels, who will be actively working among the sick, and their duty with the man wanting hospital treatment is to rush him off to a hospital without a moment's delay. The consequence is the whole machinery of the Act will be turned on the hospitals as a recruiting agency, and, therefore, I believe, gravely increase the pressure upon the beds."

In regard to out-patients, he did not think the Act would lead to any appreciable saving to the hospitals. He believed with Lord Crewe that there would be a large reduction in the number of this class of patient, but there would be an increase in the number of severe cases, and the hospitals would have to treat also those cases of special disease which cost three or four times as much as ordinary cases.

All they as hospital people had to do was to take the statement of the Chancellor as to limitation of the practice of panel doctors, and prepare to meet the requirements of those who might properly have hospital treatment.

An Interesting Scheme.

In doing that they must rely upon the Government helping them to the extent of having some provision in the Health Insurance Committee's office whereby the administrators might closely watch and keep in touch with what was going on, and if it proved that there were not enough beds in the hospitals, or there was evidence that there would not be sufficient, or that the funds would not be sufficiently liberal to enable them to go on, to be ready to deal with any emergency upon a practical basis.

There was always the bogey of State control, but it entirely rested upon what was meant by this term. His own view was that, should the Government find the hospital provision not adequate to deal with what was required for the insured people, they should go to the voluntary hospitals and say, "What beds can you provide us with for the treatment of insured persons? If you will admit these persons, and keep an account of what they cost you, we will provide that the *pro rata* cost of each such patient shall be given to the hospital."

The *pro rata* cost would not touch the voluntary principle, and would only entail the admission of Government inspectors, if it were wished, to the State wards where the insured person would be treated.

Loans Suggested.

He was perfectly certain the hospitals would want more beds, and he hoped the Government would give authority for the Treasury to make loans through a Government department for the purpose of erecting hospital buildings for the reception of patients, the loans to be based upon 3 per cent. interest, with 1 per cent. sinking fund, making 4 per cent. altogether. The repayment of this 4 per cent. to be a first charge on the patients' payments. In reality, therefore, it would mean no cost to the Exchequer. It would merely pledge the Government to provide where necessary by loan the money to meet the cost of the buildings in which these insured patients would be treated.

If the hospitals were to provide all the beds that he believed would be required to treat insured persons a very large sum indeed would be needed. The real service required from the Government was the appointment of someone constantly to watch this hospital question, and see how it could wisely be treated. Although in London hospitals the number of insured persons was 40 per cent., in the rest of England it was estimated the number would reach 50 per cent., and in Scotland even 60 per cent. of the hospital population. He congratulated them upon the latest additions to the Beckett Hospital. The wards in all respects were quite equal to, and could be compared with, the best they had in the land.

**Lord Crewe's Reply to Sir Henry Burdett.**

Lord Crewe, in reply to the vote of thanks, said he had listened with peculiar interest to the view expressed by Sir Henry Burdett in regard to the probable effect of the Act in increasing the number of in-patients in the hospitals, which differed from that which from various considerations he had ventured to put forward. It was difficult, however, successfully to look forward a year or two and say what the precise effect of a great national change of the kind would be. If Sir Henry's contention that there would be a large increase in the number of applicants for hospital treatment proved to be correct, it would point to the fact that at the present moment there was a great number of people who ought to have hospital treatment but who were now not getting it.

"I can tell you," said his Lordship, "I am certain that Sir Henry's scheme will have the very closest and most careful consideration from the department especially concerned with this subject, and ultimately, if necessary, the close attention and examination from the Government who would have to be responsible for bringing it into being. Further than this, I certainly could not go at this moment, and you would not expect me to. I listened with great interest to the scheme, and the whole tone of Sir Henry's speech was, I was particularly glad to note, of a hopeful and even sanguine character with regard to hospitals. If he and others responsible for the management of hospitals had regarded the Act in its operation as hostile to hospitals it would have been a severe blow to us, and it is gratifying to find that this hopeful view is taken."

